# JS-MA: A Jensen-Shannon Divergence Based Method for Mapping Genome-Wide Associations on Multiple Diseases

**DOI:** 10.3389/fgene.2020.507038

**Published:** 2020-10-30

**Authors:** Xuan Guo

**Affiliations:** Department of Computer Science and Engineering, University of North Texas, Denton, TX, United States

**Keywords:** GWAS, Jensen-Shannon divergence, clustering, epistasis, genetic factors

## Abstract

Taking advantage of the high-throughput genotyping technology of Single Nucleotide Polymorphism (SNP), Genome-Wide Association Studies (GWASs) have been successfully implemented for defining the relative role of genes and the environment in disease risk, assisting in enabling preventative and precision medicine. However, current multi-locus-based methods are insufficient in terms of computational cost and discrimination power to detect statistically significant interactions with different genetic effects on multifarious diseases. Statistical tests for multi-locus interactions (≥2 SNPs) raise huge analytical challenges because computational cost increases exponentially as the growth of the cardinality of SNPs in an interaction module. In this paper, we develop a simple, fast, and powerful method, named JS-MA, based on Jensen-Shannon divergence and agglomerative hierarchical clustering, to detect the genome-wide multi-locus interactions associated with multiple diseases. From the systematical simulation, JS-MA is more powerful and efficient compared with the state-of-the-art association mapping tools. JS-MA was applied to the real GWAS datasets for two common diseases, i.e., Rheumatoid Arthritis and Type 1 Diabetes. The results showed that JS-MA not only confirmed recently reported, biologically meaningful associations, but also identified novel multi-locus interactions. Therefore, we believe that JS-MA is suitable and efficient for a full-scale analysis of multi-disease-related interactions in the large GWASs.

## 1. Introduction

Genome-wide association studies (GWASs) have been proved to be a powerful tool to identify the genetic susceptibility of associations between a trait of interests using statistical tests (Sabaa et al., 2013). Recent studies have confirmed that single nucleotide polymorphisms (SNPs) are associated with a variety of common diseases (Peter and Hunter, 2009). The current primary research paradigm in GWASs is dominated by analyzing the susceptibility of single SNP to one disease at a time. One SNP might only explain a small part of causal genetic effects for multiple complex diseases (He and Lin, 2011). The word, epistasis, is defined generally as the interaction among different genes (Cordell, 2002). Many studies have demonstrated that epistasis is an important contributor to genetic variation in complex diseases. Most common diseases, such as obesity (Cordell, 2009), cancer (Ritchie et al., 2001), diabetes (Wang et al., 2012), and heart disease (Nelson et al., 2001), are complex traits, which result from a joint effect of various genetic variants, environmental factors, or their interactions. It is of great interest for us to identify the genetic risk factors for complex diseases, so as to understand disease mechanisms, develop effective treatments, and improve public health. The cost of genomic technologies is falling exponentially over time. For instance, the Human Genome Project took 13 years and cost $2.7 billion in the early twenty-first century, whereas now we can sequence a genome with $1,000 and less than a week. The availability of large-scale genotyping technology with its rapid improvement makes the cost of genome-wide analyses widely decrease, and a great number of large-scale genetic association studies are initiated. Complex diseases do not show the “simple” inheritance pattern observed in Mendelian diseases, where alterations in a single gene or a unique locus are causal for a phenotype. In complex disease, multiple genes are involved, each with low-penetrance that each gene modestly increases the probability of disease and does not ultimately determine disease status. These factors often render the traditional genetic dissection approaches, such as linkage analysis, ineffective tools to study complex diseases. In this article, we consider epistatic interactions as the statistically significant associations of *d*-SNP modules (*d* ≥ 2) with multiple phenotypes (Wang et al., 2011).

The problem of detecting high-order genome-wide epistatic interaction for case-control data has attracted more research interests recently. Generally, there are two challenges in mapping genome-wide associations for multiple diseases on a large GWAS dataset (Guo et al., 2014a): the first is arose from the heavy computational burden, i.e., the number of association patterns increases exponentially as the order of interaction goes up. For example, there are around 6.25 × 10^11^ statistical tests required to detect pairwise interactions for a moderate dataset with ~500,000 SNPs. The second challenge is that existing approaches do not have enough statistical powers to report significant high-order multi-locus interaction on multiple diseases. Because of the huge number of hypotheses and the limited sample size, a large proportion of significant associations are expected to be false positives. In recent, many computational algorithms have been proposed to overcome the above difficulties. They can be broadly classified into three categories (Xie et al., 2012): exhaustive search, stepwise search, and heuristics approach. The naive solution to tack the problem is exhaustive search using statistical tests, like χ^2^ test, exact likelihood ratio test or entropy-based test, for all SNP modules (Wan et al., 2010c; Liu et al., 2011; Yung et al., 2011). In order to minimize the huge computation requests, stepwise search strategies select a subset of SNPs or their combinations based on some low-order measurement tests, then extend them to higher-order interactions if it is statistically possible (Marchini1 et al., 2005; Li, 2008). Heuristic methods adopt machine learning or stochastic procedures to search the space of interactions rather than explicitly enumerating all combinations of SNPs (Zhang and Liu, 2007; Wan et al., 2010b). More details about the popular GWAS mapping tools can be found in recent surveys (Guo et al., 2014b; Niel et al., 2015; Visscher et al., 2017; Wen et al., 2017).

To the best of our knowledge, most epistasis detecting tools are only capable of identifying interactions on the data of GWAS with two groups, i.e., case-control studies. These tools are incompetent to discover genetic factors with diverse effects on multiple diseases. Moreover, using a limited number of case samples may lose the benefit of alleviating deficiency of statistical powers by pooling different disease samples together. Recently, Guo et al. developed a Bayesian inference based method, named DAM, to detect multi-locus epistatic interactions on multiple diseases (Guo et al., 2015, 2017). From our experiments, DAM took 3 days to finish the analyzing a real GWAS dataset using a desktop computer and only reported a few significant epistatic interactions. In this manuscript, we present a heuristic method, named JS-AM, based on Jensen-Shannon divergence and agglomerative hierarchical clustering to select a set of candidate SNPs that potentially have effects on multiple phenotypic traits (Guo, 2015). A stepwise interaction evaluation is engaged in JS-MA to further determining the association types. Systematic experiments on both simulated and real GWAS datasets demonstrate that JS-AM is feasible for identifying multi-locus interaction using GWAS datasets and enriches some novel, significant high-order epistatic interactions with various effects on multiple diseases.

## 2. Materials and Methods

### 2.1. Notation

For a GWAS dataset, let *L* denote the total number of groups, including *L* − 1 case groups and one control group. Each group has *N*_*l*_ samples with *l* ∈ {1, 2, …, *L*}. Let *N* be the total count of samples from these *L* groups, and *M* be the number of diallelic SNP markers. In general, the major alleles are represented by uppercase letters (e.g., *A*, *B*,.) and the minor alleles are represented by lowercase letters (e.g., *a*, *b*). We use {0, 1, 2} to represent {*AA, Aa, aa*}. We use *X* to indicate the SNP set, where *x*_*i*_ indicates the *i*-th SNP. Let *g*_*x*_*i*_, …, *x*_*j*__ be the combination of genotypes giving a list of SNPs {*x*_*i*_, …, *x*_*j*_}. The probability distribution of *g*_*x*_*i*_, …, *x*_*j*__ is denoted as *p*_*g*__*x*__*i*_, …, *x*_*j*___, or *p*_*g*_ for simplicity.

**Figure 1:**
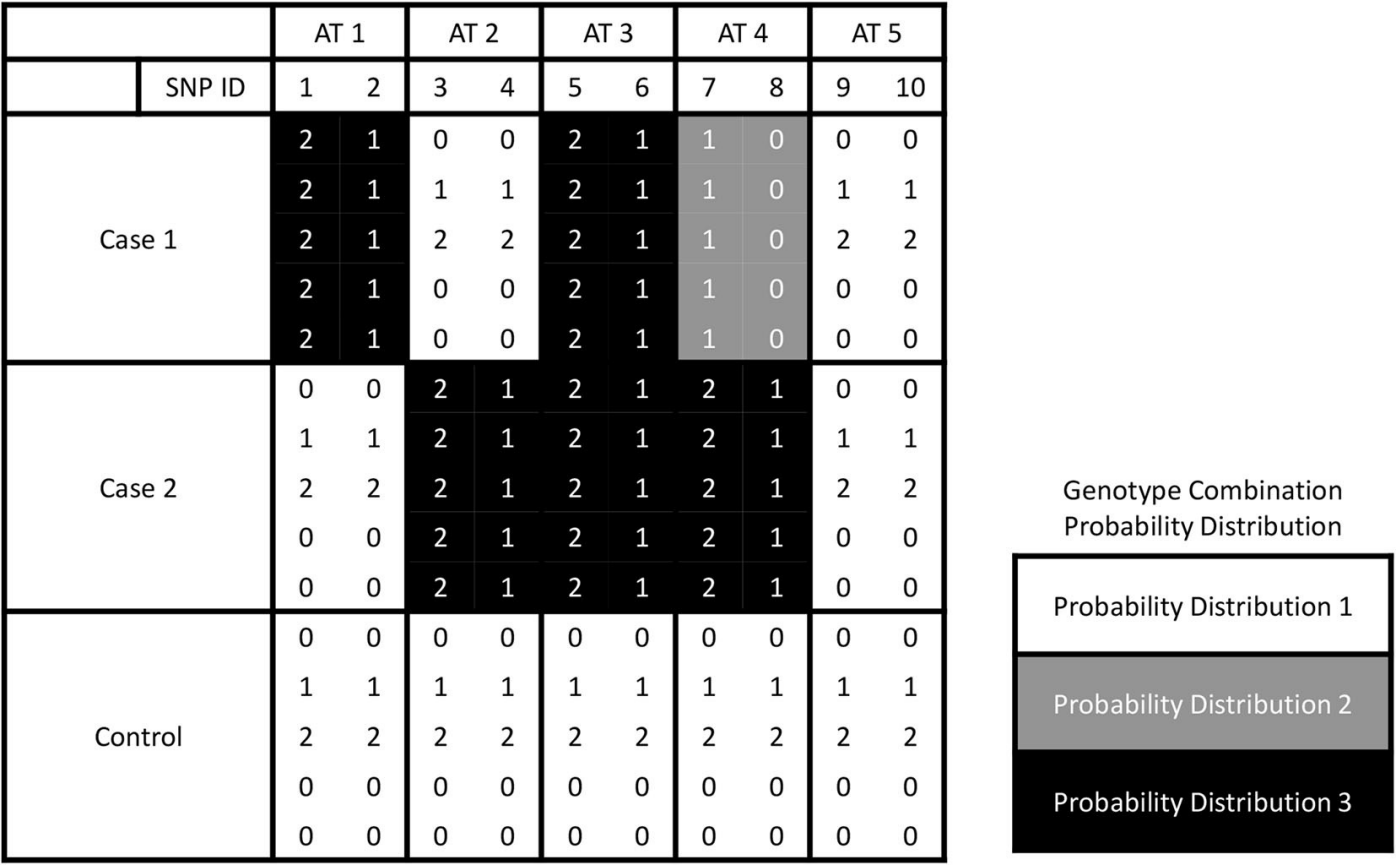
The illustration for five association types by giving three groups. Ten SNPs of AT 1, 2, 3, 4, and 5 are associated with the phenotype traits with interactions between each pair of them.

Different from the most existing methods that deal with one case and one control groups, we have two or more cases. The number of partitions of *L* groups is known as the Bell number (Guo et al., 2015). The SNPs can be assigned to be associated with one or more cases either with the same or different effects. Here, we call the assignment based on association as trait-association types, or AT in short. An example about five association types for a three-group dataset is shown in Figure 1. In this example, each AT includes 2 SNPs. There are three different probability distributions of genotype combinations, which are labeled by color white, gray, and black. SNPs 1 & 2 are related to case 1, and we call this type effect as AT1. Similarly, we call the trait-association types for SNPs 3 & 4 and SNPs 5 & 6 are AT2 and AT3, respectively. For SNPs 7 & 8, the genotype combinations display different effects on two cases, and we label it as AT4. For the last two SNPs, they are not related to any case, i.e., following the same probability distribution among three groups, and we call it AT5. In general, the number association types is increasing as the number of phenotype groups increases, which is controlled by the Bell number. We use Ψ to denote the set of association types that have different probability distribution between the case and control groups. Given *L* groups, we denote the number of all pairwise combinations as |*H*| = *L*(*L* − 1)/2 and the combination set as *H* = {*h*_1_, …, *h*_|*H*|_}. The probability distributions of genotype data in *h*_*i*_ combination are denoted as $p^{\left(h_{i}\right)}$ and $q^{\left(h_{i}\right)}$ for the first and second groups, respectively.

### 2.2. Jensen-Shannon Divergence

We used a distance measurement based on the Jensen-Shannon divergence (JS) for measuring the similarity between two SNPs. JS is a popular distance measurement based on Kullback-Leibler divergence (Lin, 1991), which evaluates the similarity between two probability distributions. Given two distributions, *p* and *q*, both with *g* categories, the Kullback-Leibler divergence is defined as follows:

(1) $$\begin{array}{l} \text{𝕂𝕃}\left(p∥q\right)=\overset{g}{\underset{i=1}{\sum }}p_{g}log\frac{p_{g}}{q_{g}} \end{array}$$

The KL divergence is not a distance because it is not symmetric. One symmetric version of KL divergence is JS, defined as:

(2) $$\begin{array}{l} JS\left(p,q\right)=0.5\text{𝕂𝕃}\left(p∥\frac{p+q}{2}\right)+0.5\text{𝕂𝕃}\left(q∥\frac{p+q}{2}\right) \end{array}$$

where $\frac{p+q}{2}$ is the pointwise mean of *p* and *q*. Here, for a genotype *g*, $\frac{p+q}{2}$ is equal to the average of *p*_*g*_ and *q*_*g*_. Given a pairwise group combination *h*_*k*_ and two SNPs, *x*_*i*_ and *x*_*j*_, we denote the probability distributions of the genotype combination of *x*_*i*_ and *x*_*j*_ as $p^{h_{k}}$ for the first group and $q^{h_{k}}$ for the second group. Based on JS, we define the distance between two SNPs, *x*_*i*_ and *x*_*j*_ as follows:

(3) $$\begin{array}{l} Dist\left(x_{i},x_{j}\right)=\frac{\sum_{h_{k}\in H}JS\left(p^{h_{k}},q^{h_{k}}\right)}{\left|H\right|} \end{array}$$

If these two SNPs are associated to any cases, the distribution of genotype combinations in case groups should be the same as the one in control. And *Dist*(*x*_*i*_, *x*_*j*_) should be a very small value toward 0; otherwise, *Dist*(*x*_*i*_, *x*_*j*_) is a large value toward 1.

### 2.3. Clustering

Our goal is to find a list of SNP modules containing *d*(*d* ≥ 2) SNPs, which have large JS dissimilarity between any two groups. It is computationally expensive to examine all *d* SNP combinations when *d* ≥ 3 given millions of SNPs in one dataset. In order to diminish the time complexity, we use agglomerative hierarchical clustering to group SNPs into clusters so that SNPs jointly affecting a trait go into separate clusters. More specifically, the complete-linkage clustering criterion was used to determine the distance between sets of SNPs. The distance from an SNP, *x*_*i*_, to a cluster, *C*, is defined as

(4) $$\begin{array}{l} Dist\left(x_{i},C\right)=\underset{x_{j}\in C}{\max}Dist\left(x_{i},x_{j}\right) \end{array}$$

The distance between two clusters is defined as

(5) $$\begin{array}{l} Dist\left(C_{i},C_{j}\right)=\underset{a\in C_{i},b\in C_{j}}{\max}Dist\left(a,b\right) \end{array}$$

In the implementation of JS-MA, we used the nearest-neighbor chain algorithm (Murtagh, 1983; Müllner, 2011). Compared to the greedy algorithm that repeatedly forms a new cluster by merging the closest pair of clusters, the nearest-neighbor chain algorithm runs faster by merging pairs of clusters in a different order. In brief, the nearest neighbor chain algorithm grows a chain of clusters, where the newly added cluster is the nearest neighbor of the previous one, and stops growing when reaching a pair of clusters that are mutual nearest neighbors. For our complete-linkage clustering criterion, the nearest neighbor chain algorithm can be guaranteed to generate the same hierarchical clustering as the greedy algorithm (Murtagh, 1983; Müllner, 2011). The time complexity of the nearest-neighbor chain algorithm is *O*(*M*^2^), where *M* is the number of SNPs. In our setting, we will stop the chain growing once the number of clusters reaches the expected number. Here, the number of clusters is a user-defined parameter. It can be set to the largest, expected size of epistatic modules. In our simulation, we set the number of clusters to two and three for 2- and 3-locus models, respectively. In the real data experiments, we set the number of clusters to ten. Once the clustering is done, top *f* SNPs from every cluster are selected for further interaction testing. Here, *f* is a user-defined number. An SNP will be picked if it shows a high dissimilarity measured by JS with other SNPs between any two groups. Every SNP is ranked based on the following score.

(6) $$\begin{array}{l} Score\left(x\right)=\underset{x\notin C_{i},}{\sum }Dist\left(x,C_{i}\right) \end{array}$$

### 2.4. Stepwise Evaluation of Interaction

We apply the χ^2^ statistic and the conditional χ^2^ test similar to the ones in (Guo et al., 2015) to measure the statistical significance for a SNP module. Let *A* = (*x*_1_, *x*_2_, …, *x*_*d*_:*T*) denote an SNP module *A* with *d* SNPs of association type *T*. We use $\chi^{2}\left(x_{1},x_{2},\ldots ,x_{d}:T\right)$ to denote the χ^2^ statistic of *A* and $\chi^{2}\left(x_{1},x_{2},\ldots ,x_{d}|x_{c_{1}},x_{c_{2}},\ldots ,x_{c_{d^{\prime }}}:T\right)$ as the conditional χ^2^ statistic given a subset ${\text{𝔸}}^{\prime }=\left(x_{c_{1}},x_{c_{2}},\ldots ,x_{c_{d^{\prime }}}\right)$ with *d*′ SNPs. The χ^2^ statistic is calculated as

(7) $$\begin{array}{l} \chi^{2}\left(x_{1},x_{2},\ldots ,x_{d}:T\right)=\overset{\left|S_{T}\right|}{\underset{i=1}{\sum }}\overset{3^{d}}{\underset{s=1}{\sum }}\frac{{\left(n_{i,s}-e_{i,s}\right)}^{2}}{e_{i,s}} \end{array}$$

where *n*_*i, s*_ is the frequency of *s*-th genotype combination in *i*-th disjoint set for the association type *T*, *e*_*i, s*_ is the corresponding expected frequency, and *S*_*T*_ denotes all the disjoint sets for *L* groups. The degrees of freedom for Equation (7) is $\left(|S_{T}|-1\right)\cdot \left(3^{d}-1\right)$. The conditional χ^2^ statistic is defined as follows

(8) $$\begin{array}{l} \chi^{2}\left(x_{1},\ldots ,x_{d}|x_{c_{1}},\ldots ,x_{c_{d^{\prime }}}:T\right)=\\ \;\overset{3^{d^{\prime }}}{\underset{\iota =1}{\sum }}\overset{\left|S_{T}\right|}{\underset{i=1}{\sum }}\overset{3^{d-d^{\prime }}}{\underset{s=1}{\sum }}\frac{{\left(n_{i,s}^{\left(\iota \right)}-e_{i,s}^{\left(\iota \right)}\right)}^{2}}{e_{i,s}^{\left(\iota \right)}} \end{array}$$

where we calculate χ^2^ statistic for 𝔸 − 𝔸′ separately for each genotype combination in 𝔸′. The degrees of freedom for Equation (8) is $3^{d^{\prime }}\cdot \left(|S_{T}|-1\right)\cdot \left(3^{d-d^{\prime }}-1\right)$. We treat SNPs as redundant SNPs when they are conditional independent given a subset of the SNP module. To avoid the redundant SNPs, we are looking for compact epistatic interactions, which is defined as follows:

**Definition 1**. *An SNP module* *A* *=* (*x*_1_, *x*_2_, *…,* *x*_*d*_) *is considered as a significant, compact interaction given a significant level α*_*d*_, *if it meets the following two conditions:*

*(1) The* p*-value of* $\chi^{2}\left(x_{1},\ldots ,x_{d}\right)\leq \alpha_{d}$, *where the* p-*value of* $\chi^{2}\left(x_{1},\ldots ,x_{d}\right)=\min_{T}\chi^{2}\left(x_{1},\ldots ,x_{d}:T\right)$;

*(2) The* p*-value of* $\chi^{2}\left(x_{1},\ldots ,x_{d}|x_{c_{1}},\ldots ,x_{c_{d^{\prime }}}\right)\leq \alpha_{d}$*, for* $\forall {\text{𝔸}}^{\prime }=\left(x_{c_{1}},x_{c_{2}},\ldots ,x_{c_{d^{\prime }}}\right)$, *given the association type* $={\mathrm{argmin}}_{T}\chi^{2}\left(x_{1},\ldots ,x_{d}:T\right)$.

Based on the Definition 1, we develop a stepwise algorithm to search for *d*-locus significant compact interactions. We assume that one SNP can only participate in one significant interaction and is only associated with one association type. We first search all modules with only one SNP based on Definition 1. Then we recursively enlarge the SNP module size by one at a time until it reaches a user pre-set value *d*. We add all novel *d*-way interactions (i.e., none of the SNPs in the module has been reported earlier) that are significant to a list *L* after applying Bonferroni correction for $\Psi \cdot \left(\begin{array}{c} M\\ d \end{array}\right)$ tests. For the interactions whose subsets have been reported as significant before, we use the conditional independent test, and put the interaction in 𝕃 if it is still significant after Bonferroni correction for $\Psi \cdot \left(\begin{array}{c} M\\ d \end{array}\right)\cdot \left(\begin{array}{c} d\\ d^{\prime } \end{array}\right)$ tests. We also apply a distance constraint that the physical distance between two SNPs in a multi-locus module should be at least 1Mb when analyzing real data. This constraint is used to avoid associations that might be due to the linkage disequilibrium effect (Cordell, 2002).

### 2.5. Algorithm

The details of the JS-MA algorithm are shown in Algorithm 1 consisting of three steps: clustering, SNP ranking, and stepwise evaluation. In clustering, the nearest neighbor chain algorithm repeatedly follows a chain of clusters, where each cluster is has the smallest distance to the previous one, until the number of clusters reaching user-defined parameter. In the second step, all SNPs are ranked based on Equation (6) and inserted into a size-limited descending list to select promising SNPs. In the last step, the χ^2^ and the conditional χ^2^ statistics are used to search for the significant, compact epistatic interactions.

## 3. Experimental Design

In this section, we introduce the simulation design, including the definitions of 10 two-locus, 6 three-locus multi-disease models and the power metric. The other start-of-the-art methods we used to compared with JS-MA, including BOOST (Wan et al., 2010a), DAM (Guo et al., 2015), SEE (Sun et al., 2019), and SNPRuler (Wan et al., 2010b). Note that BOOST and SEE are designed for detecting gene-gene interactions, i.e., interactions between two loci.

### 3.1. Data Simulation

To evaluate the performance of JS-MA, we perform extensive simulation experiments using 10 two-locus disease models (Model 1–10) and 6 three-locus models (Model 11–16) with three groups, including 2 case and 1 control groups. Since there are three phenotype groups, we could have five different association types (ATs 1–5). Note that AT1 and AT2 are equivalent if case 1 and case 2 are interchangeable, which is the case in our simulation.

The odds tables describing these 16 models are in the Supplementary Material. For the two-locus models, models 1–4 are the base models, and the rest are derived from the base ones by combining two models or letting one case group follow the same distribution as the control group. For the four two-locus base models, we took the same parameters as in Wan et al. (2010a) and Guo et al. (2014a). More specifically, we have *h*^2^ = 0.03 for Model 1, *h*^2^ = 0.02 for Models 2, 3, and 4 and *p*(*D*) = 0.1 for all four models. Minor allele frequencies (*maf*) are set to three levels: {0.1, 0.2, 0.4}. For the three-locus models, models 11 to 13 are the base models, the rest are derived using the same way as for the two-locus models. We set *h*^2^ = 0.03 and *p*(*D*) = 0.1 for Model 11, 12, and 13. The solved parameters μ and θ under different settings are provided in the Supplementary Material. The genotypes of unassociated SNP are generated by the same procedure used in previous studies (Guo et al., 2014a) with *mafs* sampled from [0.05, 0.5].

As introduced in the section 2.1, AT1 indicates the loci having different effects on the first case group compared to the other groups. AT2 indicates the loci having different effects on the second case group compared to the other groups. AT3 indicates the loci showing an identical effect on both case groups but different from the control group. AT4 indicates the loci with distinct effects on each group. We generate 100 replicas for each model, as well as for each *maf*. Note that some models do not have mathematical solution for μ and θ when *maf* = 0.1 or = 0.2. In this case, the power metric value is missing for all methods. Each simulated replica contains *M* = 1, 000 SNPs. The sample sizes of two case groups and one control group are set to (500, 500, 1, 000) or (1, 000, 1, 000, 2, 000).

### 3.2. Statistical Power

The measure of discrimination power is defined as the fraction of 100 replicas on which the ground-truth associations are the top one signification epistatic interactions.

## 4. Results and Discussion

In this section, we first present the type 1 error rate of JS-MA under the null model. And then we show the experimental results on the simulated datasets. We also present the results of JS-MA on two real GWAS datasets from WTCCC (Zeggini et al., 2007), i.e., Rheumatoid Arthritis (RA) and Type 1 Diabetes (T1D). Note that among these five approaches, only JS-MA and DAM are able to label the association types that we defined in section 2.1, and the rest methods can only report the interactions without information about the phenotype(s) on which they have genetic effects.

### 4.1. Null Simulation to Test Type I Errors

**Figure 2:**
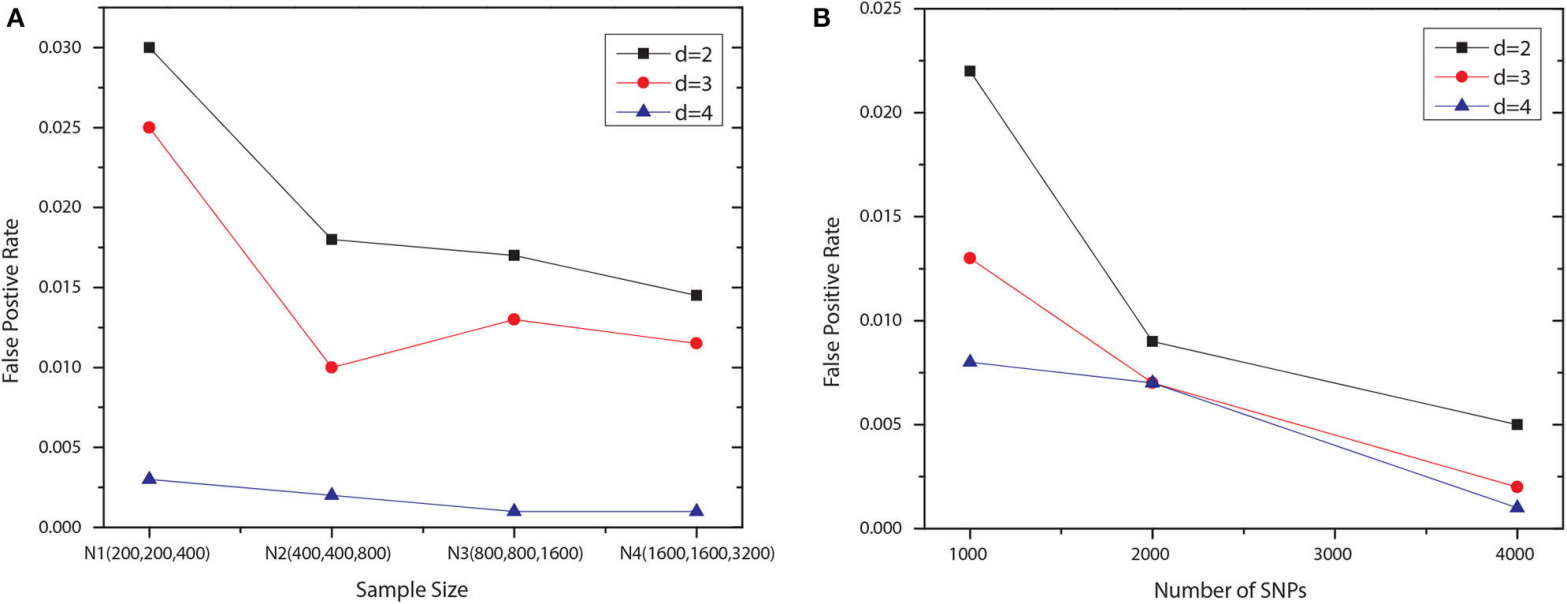
False positive rates of JS-MA under null simulation. The plots in **(A,B)** show the false positive rates for different *d*s, sample sizes, and the numbers of SNP.

We examined the type I error rate for interactions with different number of SNPs, i.e., *d* = 2, 3, 4. We generated 1,000 null datasets for six settings, respectively. Specifically, we fixed the number of SNP to 1,000 and vary the number of samples in each group. The first four settings contained the following numbers of samples: *N*1 = {200, 200, 400}, *N*2 = {400, 400, 800}, *N*3 = {800, 800, 1, 600}, and *N*4 = {1, 600, 1, 600, 3, 200}, where the first two numbers indicated the sizes of two case groups, and the last number was the control group size. For the last two settings, using *N*4, we increased the number of SNP to 2,000 and 4,000. All SNPs were generated independently, with *maf* uniformly distributed in [0.05, 0.5]. Note that we set the significance level to 0.1 and applied the Bonferroni correction for multiple hypothesis testing. The degree of freedom for Pearson's χ^2^ test is *df* = (|*T*|−1)(|*G*|−1), where |*T*| denotes the number of disjoint set of groups for the association type |*T*|, and *G* is the set of genotypes given the SNP module. The degree of freedom for conditional χ^2^ test is |*G*′|(|*T*|−1)(|*G*/*G*′|−1), where *G*′ is the set of genotypes given a subset of the SNP module, and *G*/*G*′ denotes the set of genotypes for the rest SNPs. The results shown in Figure 2 demonstrated that JS-MA can well control the type I error rate.

### 4.2. Simulation Experiments on Two-Locus Models

**Figure 3:**
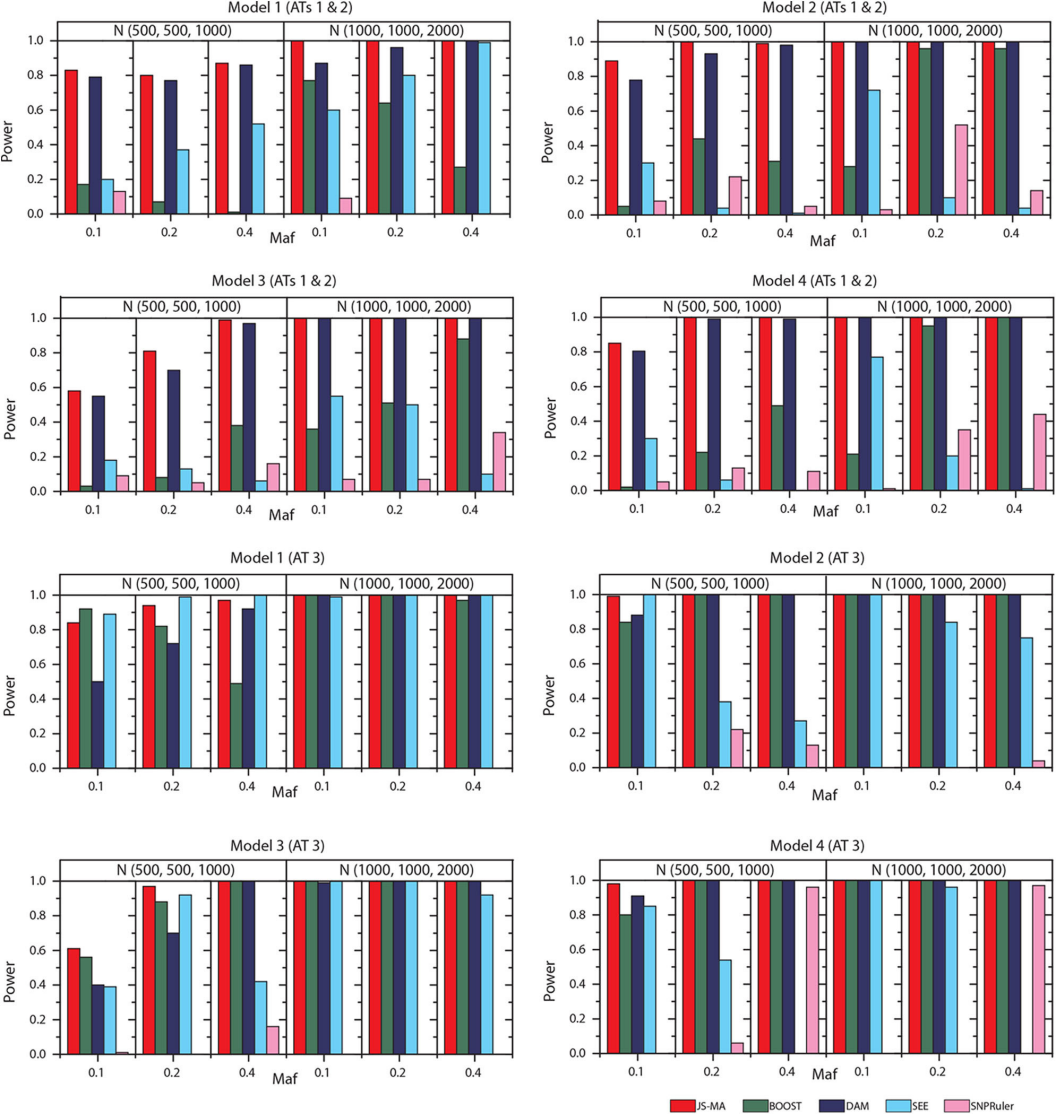
Performance comparison between JS-AM, BOOST, DAM, SEE, and SNPRuler on the simulated two-locus models 1, 2, 3, and 4 for association types 1, 2, and 3.

**Figure 4:**
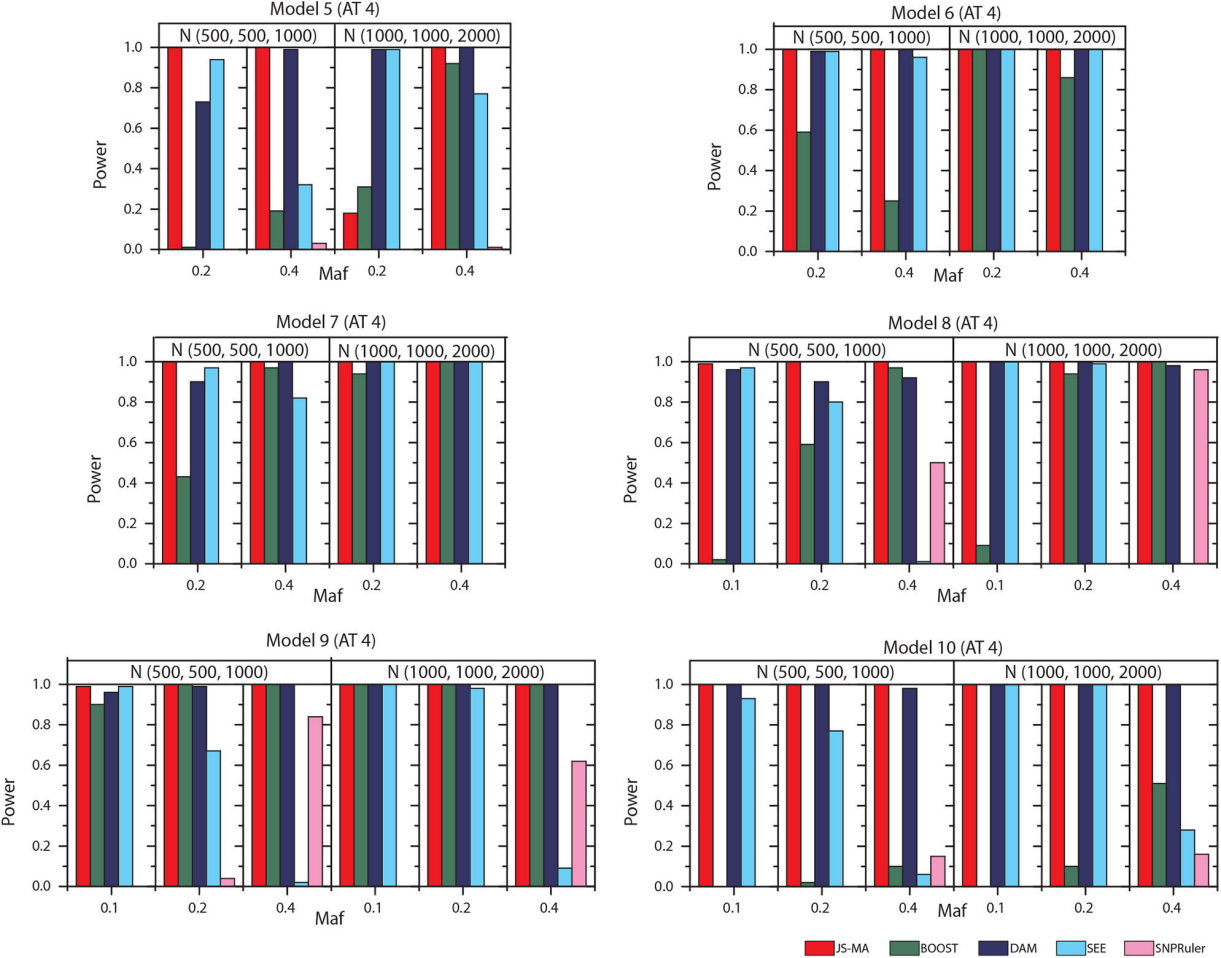
Performance comparison between JS-AM, BOOST, DAM, SEE, and SNPRuler on the simulated two-locus models 5–10 for association type 4. Note that the models 5, 6, and 7 have no mathematical solution when *maf* = 0.1.

We tested the performance of JS-MA and four other methods on the datasets generated by two-locus models. The test results are illustrated in Figures 3, 4. As we expected, the powers of all methods increased when the sample size increased from (500, 500, 1,000) to (1,000, 1,000, 2,000). For all models, the powers of JS-MA and SEE increased when the *maf* increased from 0.1 to 0.4. We do not observe a similar trend for BOOST, DAM, and SNPRuler. All models were more powerful for AT3 than ATs 1 and 2 because ATs 1 and 2 have some cases similar to controls, which makes it hard to locate the embedded interactions. Overall, the powers of JS-MA are higher compared to other methods except in a few cases where the power is comparable with others. For a more intuitive comparison, we adopt a concept, overall quality *q* = 100 × *n*_*correct*_/*n*_*total*_ from (Guo et al., 2014a), where *n*_*correct*_ is the number of datasets from which the method successfully detected the ground-truth interaction, and *n*_*total*_ is the total number of datasets. The overall quality of JS-MA, BOOST, DAM, SEE, and SNPRuler are 94, 50, 89, 51, and 11% for the sample size (500, 500, 1, 000), and 97, 78, 93, 71, and 13% for the sample size (1, 000, 1, 000, 2, 000), respectively. It showed that JS-MA achieved 3–5% better results than the second best.

### 4.3. Simulation Experiments on Three-Locus Models

**Figure 5:**
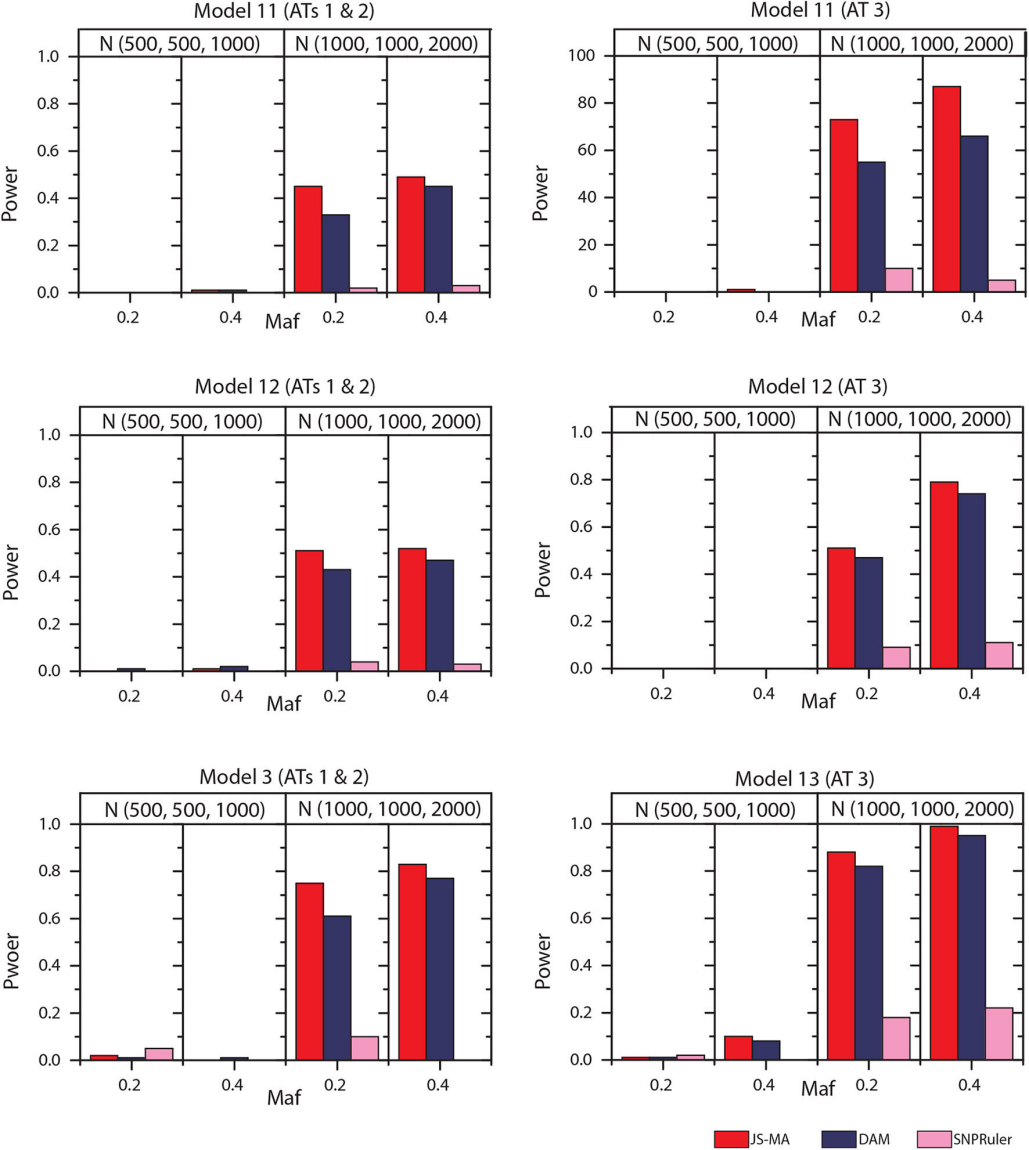
Performance comparison between JS-AM, DAM, and SNPRuler on the simulated three-locus models 11, 12, and 13 for association types 1, 2, and 3.

**Figure 6:**
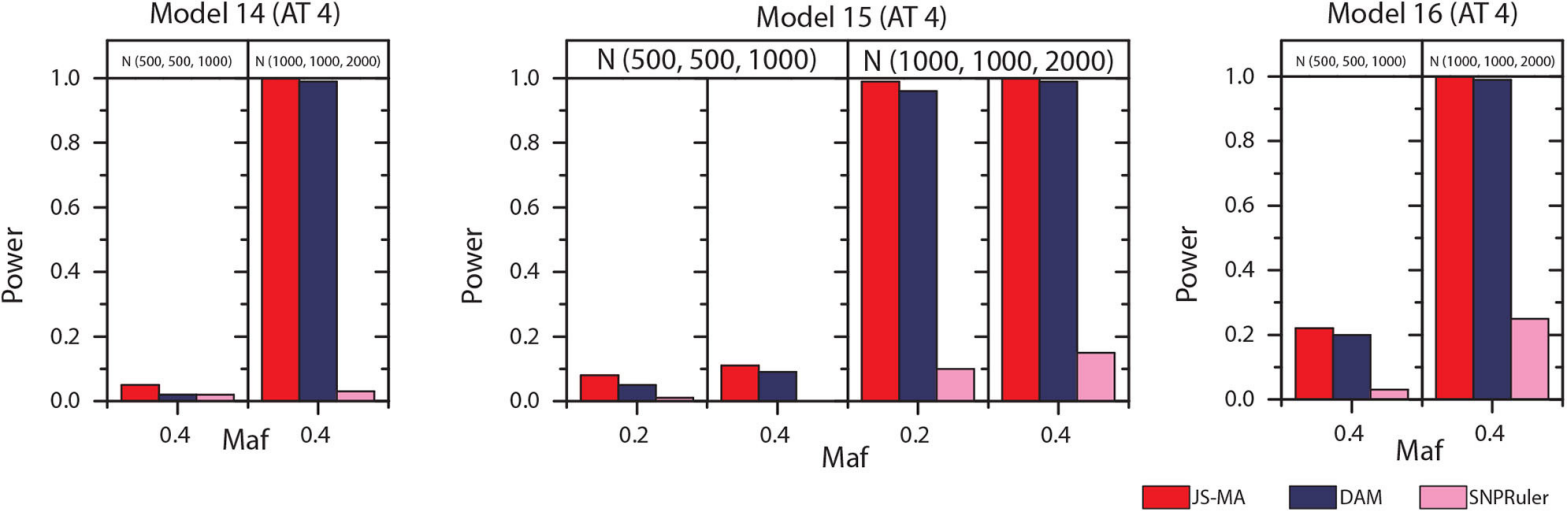
Performance comparison between JS-AM, DAM, and SNPRuler on the simulated three-locus models 14, 15, and 16 for association type 4. Note that the models 14 and 16 have no mathematical solution when *maf* = 0.2.

The experimental results on models 11–16 are shown in Figures 5, 6. In these experiments, BOOST and SEE were dropped because they cannot detect three-locus interactions. From Figures 5, 6 we can find that all three methods had nearly no power when the sample size is small. It is reasonable since a high-order interaction needs to have larger effect size for small sample size compared to large sample size. When the sample size was doubled, all three methods started to gain some power. Compared to the results from two-locus models, all the methods are not as powerful as before. In all settings, JS-MA is the most powerful approach. Using the same overall quality measurement introduced in the last section, JS-MA, DAM, and SNPRuler reached 4, 3, and 1% for sample size (500, 500, 1,000), and 77, 70, and 9% for sample size (1,000, 1,000, 2,000), respectively.

### 4.4. Computation Efficiency

From a practical point of view, a challenging bottleneck of mapping multi-locus epistatic interactions in GWASs is the computational efficiency. Traditional tools for two-locus epistatic interaction detection usually take several days for a dataset with millions of SNPs using a standard desktop (Wan et al., 2010a). We measured the running time of JS-MA, BOOST, DAM, SEE, and SNPRuler on one computing node of an HPC system with a UNIX operating system, Intel Xeon E5-2699v4 Broadwell, and 128 GB memory. The results are shown in Table 1. Here, we set the target number of SNPs in an epistatic interaction to be two, and the rest of the parameters for each tool were left unchanged with default values. Table 1 showed that JS-MA was faster than BOOST, DAM, and SNPRuler in most scenarios. The running time used by JS-MA did not increase as fast as SNPRuler and DAM did when the number of SNPs increased. Since SEE is a heuristic method, it used the least amount of time. However, its performance is not as good as the rest. We also measured the memory consumption for JS-MA. JS-MA used 10, 62, and 130 MB for 1,000, 5,000, and 10,000 SNPs, respectively. The majority of the consumed memory was used for storing the genotype data.

**Table 1:** Time comparison of JS-MA, BOOST, DAM, SEE, and SNPRuler (in seconds).

**Data size**	**JS-MA**	**BOOST**	**DAM**	**SEE**	**SNPRuler**
*N* = 6,000, *M* = 1,000	8	6	31	6	13
*N* = 6,000, *M* = 5,000	20	31	187	10	184
*N* = 6,000, *M* = 10,000	81	96	512	18	741

### 4.5. Experiments on The WTCCC Data

We employed JS-MA to analyze real data from the WTCCC Zeggini et al. (2007) for two common human diseases, i.e., Rheumatoid Arthritis (RA), Type 1 Diabetes (T1D). There are 3999 cases and 3004 shared controls. We constructed a dataset with RA as case 1 and T1D as case 2. The procedure of quality control is the same as presented in Guo et al. (2014a). After the SNP filtration, the dataset contains 333,739 high-quality SNPs. By setting *f* × *k* = 100 with *k* = 10 as the number of clusters, JS-MA finished the searching in 3 h using the same computing node, which was used in the computation time analysis. JS-MA reported some novel epistatic interactions. For example, (rs6679677, rs805301) was labeled as AT4, and its *p*-value is 6.2 × 10^−120^ from the χ^2^ test. For this interaction, rs6679677, located on Chromosome 1, has been reported to be associated with both RA and T1D (Burton et al., 2007). The association between rs6679677 and T1D is due to a closely linked, potentially causal variant identified as rs2476601, which is also known as Arg620Trp (Smyth et al., 2008). Whereas, rs805301 is located inside gene BAG6 on Chromosome 6. BAG6 encodes a nuclear protein that forms a complex with E1A binding protein p300 and is required for the response to DNA damage. The SNP module (rs6679677, rs805301) shows different association effects on RA and T1D compared to the control group. Another interesting interaction is (rs200991, rs11171739) labeled as AT2, and its *p*-value is 6.7 × 10^−26^ from the χ^2^ test. In this interaction, rs200991 is located on Chromosome 6 near the gene, HIST1H2BN, which encodes Histone H2B type 1-N. Histones play a central role in transcription regulation, DNA repair, DNA replication, and chromosomal stability. And rs11171739 has been reported to be associated with T1D (Burton et al., 2007). AT2 means the SNP module may not have a genetic effect on RA.

**Algorithm 1:** The JS-MA Algorithm.

**Require:** An *N* × (*M* + 1) matrix
**Require:** Number of clusters *k*, top *f* SNPs in a cluster
1: Read *N* × (*M* + 1) matrix file
2: Calculate the pairwise distance based on JS (Equation 3)
3: Initialize each SNP as a cluster
4: *n* ← *M*
5: **while** *n* > *k* **do**
6: Apply nearest neighbor chain algorithm
7: *n* − = 1
8: **end while**
9: Initialize descending list *L* with length *f* × *k*
10: **for** each SNP *x* **do**
11: Calculate *Score*(*x*)
12: Place *x* into 𝕃 if *Score*(*x*) is among top *f* SNPs
13: **end for**
14: Stepwise evaluate all possible SNP modules using SNPs in *L*

JS-MA also reported some three-locus epistatic interactions. For instance, (rs6679677, rs377763, rs9273363) labeled as AT2 with *p*-value 1.3 × 10^−116^. Both rs377763 and rs9273363 are located on Chromosome 6. rs377763 is near the downstream of gene NOTCH4, which is found to be associated with multiple sclerosis, a chronic inflammatory disease. rs9273363 is inside the gene HLA-DQA1, which plays a critical role in the immune system. The protein produced from the HLA-DQA1 gene binds to the protein produced from the MHC class II gene, HLA-DQB2. Many studies have reported the MHC region on chromosome 6 with respect to infection, inflammation, autoimmunity, and transplant medicine (Lechler and Warrens, 2000; Wan et al., 2010a; Zhang et al., 2012). A four-locus interaction found by JS-MA is (rs10924239, rs17432869, rs7610077, rs11098422) labeled as AT4 with *p*-value 3.9 × 10^−106^. rs10924239 is an intron variant of the gene KIF26B on Chromosome 1. KIF26B is essential for embryonic kidney development. rs17432869 is located on Chromosome 2 and inside gene LOC105373439, which is an RNA Gene and is affiliated with the ncRNA class. rs7610077 is located on Chromosome 3 and inside gene SNX4, which encodes a member of the sorting nexin family. rs11098422 is located on Chromosome 4 and inside gene NDST3, whose expression impacts the cardiovascular system. Validating the relationship between these SNP modules and RA and T1D is beyond the scope of this work. The significant enrichment of some genotype combinations from these SNP modules in both cases implies that they might interact and/or be associated with these two diseases.

## 5. Conclusion

The enormous number of SNPs genotyped in genome-wide case-control studies poses a significant computational challenge in the identification of gene-gene interactions. During the last few years, many computational and statistical tools are developed to find gene-gene interactions for the data containing only two traits, i.e., case-control groups. Here, we present a novel method, named “JS-MA,” to address the computation and statistical power issues in multi-disease GWASs. We have successfully applied JS-MA to systematically simulated datasets and analyzed two real GWAS datasets. Our experimental results on both simulated and real data demonstrate that JS-MA is capable of detecting high-order epistatic interactions for multiple diseases at the genome-wide scale. It is worth mentioning that when JS-MA is used to analyze real data, quality control procedures are necessary because sequencing bias and genotyping bias could confound JS-MA by leading to false-positives. For example, the coverage bias caused by sequencing machines may have SNPs with low, uneven coverage. Thus, quality control is required to filter out unreliable SNPs.

## Data Availability Statement

The raw data supporting the conclusions of this article will be made available by the authors, without undue reservation, to any qualified researcher.

## Author Contributions

XG designed, implemented, and tested the proposed methods.

## Conflict of Interest

The author declares that the research was conducted in the absence of any commercial or financial relationships that could be construed as a potential conflict of interest.
