# Supplementary material for: JS-MA: A Jensen-Shannon Divergence Based Method for Mapping Genome-Wide Associations on Multiple Diseases
Source: Front Genet. 2020 Oct 30;11:507038. doi: 10.3389/fgene.2020.507038 (PMC7662082; doi:10.3389/fgene.2020.507038)
Supplement: Supplementary file 1 [file Data_Sheet_1.PDF]

Supplemental Data

Table 1. Odds table for two-locus model 1, 2, 3 and 4.

Model 1	BB	Bb	bb
AA	μ	μ	μ
Aa	μ	$\mu(1+\theta)$	$\mu(1+\theta)^3$
aa	μ	$\mu(1+\theta)^3$	$\mu(1+\theta)^4$
**Model 2**	**BB**	**Bb**	**bb**
AA	μ	$\mu(1+\theta)$	$\mu(1+\theta)$
Aa	$\mu(1+\theta)$	μ	μ
aa	$\mu(1+\theta)$	μ	μ
**Model 3**	**BB**	**Bb**	**bb**
AA	μ	μ	$\mu(1+\theta)$
Aa	μ	$\mu(1+\theta)$	μ
aa	$\mu(1+\theta)$	μ	μ
**Model 4**	**BB**	**Bb**	**bb**
AA	μ	$\mu(1+\theta)$	μ
Aa	$\mu(1+\theta)$	μ	$\mu(1+\theta)$
aa	μ	$\mu(1+\theta)$	μ

Table 2: Numerical solutions for Table 1.

Model 1	$p(D) = 0.1$, $h^2 = 0.03$		
MAF	0.1	0.2	0.4
μ	0.100	0.091	0.075
θ	3.448	1.300	0.624
Model 2	$p(D) = 0.1$, $h^2 = 0.02$		
MAF	0.1	0.2	0.4
μ	0.077	0.064	0.065
θ	1.532	1.700	1.640
Model 3	$p(D) = 0.1$, $h^2 = 0.02$		
MAF	0.1	0.2	0.4
μ	0.0996	0.0893	0.0743
θ	3.0151	1.7876	1.5315
Model 4	$p(D) = 0.1$, $h^2 = 0.02$		
MAF	0.1	0.2	0.4
μ	0.078	0.067	0.061
θ	1.537	1.601	1.717

Table 3: Two-locus models with different odds patterns of two loci, the prevalence $p(D) = 0.1$ and the heritability $h^2 = 0.02$, MAF = 0.1, 0.2, 0.4.

Model 5 (mix of Model 1 and Model 2)		
Genotype	Odds of disease 1	Odds of disease 2
AABB	μ_1	μ_2
AABb	μ_1	$\mu_2(1+\theta)$
AAbb	μ_1	$\mu_2(1+\theta)$
AaBB	μ_1	$\mu_2(1+\theta)$
AaBb	$\mu_1(1+\theta_1)$	μ_2
Aabb	$\mu_1(1+\theta_1)^2$	μ_2
aaBB	μ_1	$\mu_2(1+\theta)$
aaBb	$\mu_1(1+\theta_1)^2$	μ_2
aabb	$\mu_1(1+\theta_1)^4$	μ_2

Model 6 (mix of Model 1 and Model 3)		
Genotype	Odds of disease 1	Odds of disease 2
AABB	μ_1	μ_2
AABb	μ_1	μ_2
AAbb	μ_1	$\mu_2(1+\theta)$
AaBB	μ_1	μ_2
AaBb	$\mu_1(1+\theta_1)$	$\mu_2(1+\theta)$
Aabb	$\mu_1(1+\theta_1)^2$	μ_2
aaBB	μ_1	$\mu_2(1+\theta)$
aaBb	$\mu_1(1+\theta_1)^2$	μ_2
aabb	$\mu_1(1+\theta_1)^4$	μ_2

Model 7 (mix of Model 1 and Model 4)		
Genotype	Odds of disease 1	Odds of disease 2
AABB	μ_1	μ_2
AABb	μ_1	$\mu_2(1+\theta)$
AAbb	μ_1	μ_2
AaBB	μ_1	$\mu_2(1+\theta)$
AaBb	$\mu_1(1+\theta_1)$	μ_2
Aabb	$\mu_1(1+\theta_1)^2$	$\mu_2(1+\theta)$
aaBB	μ_1	μ_2
aaBb	$\mu_1(1+\theta_1)^2$	$\mu_2(1+\theta)$
aabb	$\mu_1(1+\theta_1)^4$	μ_2

Model 8 (mix of Model 2 and Model 3)

Genotype	Odds of disease 1	Odds of disease 2
AABB	μ_2	μ_2
AABb	$\mu_2(1+\theta)$	μ_2
AAbb	$\mu_2(1+\theta)$	$\mu_2(1+\theta)$
AaBB	$\mu_2(1+\theta)$	μ_2
AaBb	μ_2	$\mu_2(1+\theta)$
Aabb	μ_2	μ_2
aaBB	$\mu_2(1+\theta)$	$\mu_2(1+\theta)$
aaBb	μ_2	μ_2
aabb	μ_2	μ_2

Model 9 (mix of Model 2 and Model 4)		
Genotype	Odds of disease 1	Odds of disease 2
AABB	μ_2	μ_2
AABb	$\mu_2(1+\theta)$	$\mu_2(1+\theta)$
AAbb	$\mu_2(1+\theta)$	μ_2
AaBB	$\mu_2(1+\theta)$	$\mu_2(1+\theta)$
AaBb	μ_2	μ_2
Aabb	μ_2	$\mu_2(1+\theta)$
aaBB	$\mu_2(1+\theta)$	μ_2
aaBb	μ_2	$\mu_2(1+\theta)$
aabb	μ_2	μ_2

Model 10 (mix of Model 3 and Model 4)		
Genotype	Odds of disease 1	Odds of disease 2
AABB	μ_2	μ_2
AABb	μ_2	$\mu_2(1+\theta)$
AAbb	$\mu_2(1+\theta)$	μ_2
AaBB	μ_2	$\mu_2(1+\theta)$
AaBb	$\mu_2(1+\theta)$	μ_2
Aabb	μ_2	$\mu_2(1+\theta)$
aaBB	$\mu_2(1+\theta)$	μ_2
aaBb	μ_2	$\mu_2(1+\theta)$
aabb	μ_2	μ_2

Table 4. Odds table for three-locus models 11, 12, and 13. Model 11 with multiplicative effect between and within loci, Model 12 with multiplicative effect between loci, and Model 13 with threshold effect

**(11)**	**BBCC**	**BBCc**	**BBcc**	**BbCC**	**BbCc**	**Bbcc**	**bbCC**	**bbCc**	**bbcc**
**AA**	μ	$\mu(1+\theta)$	$\mu(1+\theta)^2$	$\mu(1+\theta)$	$\mu(1+\theta)^2$	$\mu(1+\theta)^3$	$\mu(1+\theta)^2$	$\mu(1+\theta)^3$	$\mu(1+\theta)^4$
**Aa**	$\mu(1+\theta)$	$\mu(1+\theta)^2$	$\mu(1+\theta)^3$	$\mu(1+\theta)^2$	$\mu(1+\theta)^3$	$\mu(1+\theta)^4$	$\mu(1+\theta)^3$	$\mu(1+\theta)^4$	$\mu(1+\theta)^5$
**aa**	$\mu(1+\theta)^2$	$\mu(1+\theta)^3$	$\mu(1+\theta)^4$	$\mu(1+\theta)^3$	$\mu(1+\theta)^4$	$\mu(1+\theta)^5$	$\mu(1+\theta)^4$	$\mu(1+\theta)^5$	$\mu(1+\theta)^6$
**(12)**	**BBCC**	**BBCc**	**BBcc**	**BbCC**	**BbCc**	**Bbcc**	**bbCC**	**bbCc**	**bbcc**
**AA**	μ	μ	μ	μ	μ	μ	μ	μ	μ
**Aa**	μ	μ	μ	μ	$\mu(1+\theta)^1$	$\mu(1+\theta)^2$	μ	$\mu(1+\theta)^2$	$\mu(1+\theta)^4$
**aa**	μ	μ	μ	μ	$\mu(1+\theta)^2$	$\mu(1+\theta)^4$	μ	$\mu(1+\theta)^4$	$\mu(1+\theta)^7$
**(13)**	**BBCC**	**BBCc**	**BBcc**	**BbCC**	**BbCc**	**Bbcc**	**bbCC**	**bbCc**	**bbcc**
**AA**	μ	μ	μ	μ	μ	μ	μ	μ	μ
**Aa**	μ	μ	μ	μ	$\mu(1+\theta)$	$\mu(1+\theta)$	μ	$\mu(1+\theta)$	$\mu(1+\theta)$
**aa**	μ	μ	μ	μ	$\mu(1+\theta)$	$\mu(1+\theta)$	μ	$\mu(1+\theta)$	$\mu(1+\theta)$

Table 5: Numerical solutions for Table 4.

Model 11	$p(D) = 0.1$, $h^2 = 0.03$	
MAF	0.2	0.4
μ	0.053	0.032
θ	0.690	0.592
Model 12	$p(D) = 0.1$, $h^2 = 0.03$	
MAF	0.2	0.4
μ	0.100	0.085
θ	1.719	0.462
Model 13	$p(D) = 0.1$, $h^2 = 0.03$	
MAF	0.2	0.4
μ	0.097	0.074
θ	4.187	2.108

Table 6: Three-locus models with different odds patterns of three loci, the prevalence $p(D) = 0.1$ and the heritability $h^2 = 0.03$, MAF = 0.2, 0.4.

Model 14 (mix of Model 11 and Model 12)		
Genotype	Odds of disease 1	Odds of disease 2
AABBCC	μ_1	μ_2
AABBCc	$\mu_1(1+\theta_1)$	μ_2
AABBcc	$\mu_1(1+\theta_1)^2$	μ_2
AABbCC	$\mu_1(1+\theta_1)$	μ_2
AABbCc	$\mu_1(1+\theta_1)^2$	μ_2
AABbcc	$\mu_1(1+\theta_1)^3$	μ_2
AAbbCC	$\mu_1(1+\theta_1)^2$	μ_2
AAbbCc	$\mu_1(1+\theta_1)^3$	μ_2
AAbbcc	$\mu_1(1+\theta_1)^4$	μ_2
AaBBCC	$\mu_1(1+\theta_1)$	μ_2
AaBBCc	$\mu_1(1+\theta_1)^2$	μ_2
AaBBcc	$\mu_1(1+\theta_1)^3$	μ_2
AaBbCC	$\mu_1(1+\theta_1)^2$	μ_2
AaBbCc	$\mu_1(1+\theta_1)^3$	$\mu_2(1+\theta_2)$
AaBbcc	$\mu_1(1+\theta_1)^4$	$\mu_2(1+\theta_2)^2$
AabbCC	$\mu_1(1+\theta_1)^3$	μ_2
AabbCc	$\mu_1(1+\theta_1)^4$	$\mu_2(1+\theta_2)^2$
Aabbcc	$\mu_1(1+\theta_1)^5$	$\mu_2(1+\theta_2)^4$
aaBBCC	$\mu_1(1+\theta_1)^2$	μ_2
aaBBCc	$\mu_1(1+\theta_1)^3$	μ_2
aaBBcc	$\mu_1(1+\theta_1)^4$	μ_2
aaBbCC	$\mu_1(1+\theta_1)^3$	μ_2
aaBbCc	$\mu_1(1+\theta_1)^4$	$\mu_2(1+\theta_2)^2$
aaBbcc	$\mu_1(1+\theta_1)^5$	$\mu_2(1+\theta_2)^4$
aabbCC	$\mu_1(1+\theta_1)^4$	μ_2
aabbCc	$\mu_1(1+\theta_1)^5$	$\mu_2(1+\theta_2)^4$
aabbcc	$\mu_1(1+\theta_1)^6$	$\mu_2(1+\theta_2)^7$

Model 15 (mix of Model 11 and Model 13)		
Genotype	Odds of disease 1	Odds of disease 2
AABBCC	μ_1	μ_2
AABBCc	$\mu_1(1+\theta_1)$	μ_2
AABBcc	$\mu_1(1+\theta_1)^2$	μ_2
AABbCC	$\mu_1(1+\theta_1)$	μ_2
AABbCc	$\mu_1(1+\theta_1)^2$	μ_2
AABbcc	$\mu_1(1+\theta_1)^3$	μ_2
AAbbCC	$\mu_1(1+\theta_1)^2$	μ_2
AAbbCc	$\mu_1(1+\theta_1)^3$	μ_2
AAbbcc	$\mu_1(1+\theta_1)^4$	μ_2
AaBBCC	$\mu_1(1+\theta_1)$	μ_2
AaBBCc	$\mu_1(1+\theta_1)^2$	μ_2

AaBBcc	$\mu_1(1+\theta_1)^3$	μ_2
AaBbCC	$\mu_1(1+\theta_1)^2$	μ_2
AaBbCc	$\mu_1(1+\theta_1)^3$	$\mu_2(1+\theta_2)$
AaBbcc	$\mu_1(1+\theta_1)^4$	$\mu_2(1+\theta_2)$
AabbCC	$\mu_1(1+\theta_1)^3$	μ_2
AabbCc	$\mu_1(1+\theta_1)^4$	$\mu_2(1+\theta_2)$
Aabbcc	$\mu_1(1+\theta_1)^5$	$\mu_2(1+\theta_2)$
aaBBCC	$\mu_1(1+\theta_1)^2$	μ_2
aaBBCc	$\mu_1(1+\theta_1)^3$	μ_2
aaBBcc	$\mu_1(1+\theta_1)^4$	μ_2
aaBbCC	$\mu_1(1+\theta_1)^3$	μ_2
aaBbCc	$\mu_1(1+\theta_1)^4$	$\mu_2(1+\theta_2)$
aaBbcc	$\mu_1(1+\theta_1)^5$	$\mu_2(1+\theta_2)$
aabbCC	$\mu_1(1+\theta_1)^4$	μ_2
aabbCc	$\mu_1(1+\theta_1)^5$	$\mu_2(1+\theta_2)$
aabbcc	$\mu_1(1+\theta_1)^6$	$\mu_2(1+\theta_2)$

Model 16 (mix of Model 12 and Model 13)		
Genotype	Odds of disease 1	Odds of disease 2
AABBCC	μ_1	μ_2
AABBCc	μ_1	μ_2
AABBcc	μ_1	μ_2
AABbCC	μ_1	μ_2
AABbCc	μ_1	μ_2
AABbcc	μ_1	μ_2
AAbbCC	μ_1	μ_2
AAbbCc	μ_1	μ_2
AAbbcc	μ_1	μ_2
AaBBCC	μ_1	μ_2
AaBBCc	μ_1	μ_2
AaBBcc	μ_1	μ_2
AaBbCC	μ_1	μ_2
AaBbCc	$\mu_1(1+\theta_1)$	$\mu_2(1+\theta_2)$
AaBbcc	$\mu_1(1+\theta_1)^2$	$\mu_2(1+\theta_2)$
AabbCC	μ_1	μ_2
AabbCc	$\mu_1(1+\theta_1)^2$	$\mu_2(1+\theta_2)$
Aabbcc	$\mu_1(1+\theta_1)^4$	$\mu_2(1+\theta_2)$
aaBBCC	μ_1	μ_2
aaBBCc	μ_1	μ_2
aaBBcc	μ_1	μ_2
aaBbCC	μ_1	μ_2
aaBbCc	$\mu_1(1+\theta_1)^2$	$\mu_2(1+\theta_2)$
aaBbcc	$\mu_1(1+\theta_1)^4$	$\mu_2(1+\theta_2)$
aabbCC	μ_1	μ_2
aabbCc	$\mu_1(1+\theta_1)^4$	$\mu_2(1+\theta_2)$
aabbcc	$\mu_1(1+\theta_1)^7$	$\mu_2(1+\theta_2)$
